# Long non‐coding RNAs as key regulators of neurodegenerative protein aggregation

**DOI:** 10.1002/alz.14498

**Published:** 2025-02-12

**Authors:** Qi Xu, Dan Liu, Ling‐Qiang Zhu, Ying Su, He‐Zhou Huang

**Affiliations:** ^1^ Department of Neurology Union Hospital Huazhong University of Science and Technology Wuhan China; ^2^ Department of Genetics School of Basic Medicine Tongji Medical College, Huazhong University of Science and Technology Wuhan Hubei China; ^3^ Department of Pathophysiology School of Basic Medicine Tongji Medical College, Huazhong University of Science and Technology Wuhan Hubei China; ^4^ Department of Anesthesiology Department Union Hospital, Huazhong University of Science and Technology Wuhan China

**Keywords:** long non‐coding RNA (lncRNA), neurodegenerative disease, protein aggregation

## Abstract

The characteristic events in neurodegenerative diseases (NDDs) encompass protein misfolding, aggregation, accumulation, and their related cellular dysfunction, synaptic function loss. While distinct proteins are implicated in the pathological processes of different NDDs, the process of protein misfolding and aggregation remains notably similar across various conditions. Specifically, proteins undergo misfolding into beta‐folded (β‐folded) conformation, resulting in the formation of insoluble amyloid proteins. Despite advancements in comprehending protein aggregation, certain facets of this intricate process remain incompletely elucidated. In recent years, the concept that long non‐coding RNAs (lncRNAs) contribute to protein aggregation has gained recognition. LncRNAs influence the formation of protein aggregates by facilitating protein overexpression through the regulation of gene transcription and translation, inhibiting protein degradation via lysosomal and autophagic pathways, and targeting aberrant modifications and phase transitions of proteins. A better understanding of the relationship between lncRNAs and aberrant protein aggregation is an important step in dissecting the underlying molecular mechanisms and will contribute to the discovery of new therapeutic targets and strategies.

**Highlights:**

- NDDs are marked by protein misfolding, aggregation, and accumulation, leading to cellular dysfunction and loss of synaptic function.
- Despite different proteins being involved in various NDDs, the process of misfolding into β‐folded conformations and forming insoluble amyloid proteins is consistent across conditions.
- The role of lncRNAs in protein aggregation has gained attention, as they regulate gene transcription and translation, inhibit protein degradation, and target aberrant protein modifications.
- Understanding the link between lncRNAs and protein aggregation is crucial for uncovering molecular mechanisms and developing new therapeutic targets.

## 1 BRIEF INTRODUCTION ABOUT THE NEURODEGENERATIVE PROTEIN AGGREGATION

Neurodegenerative diseases (NDDs) denote the progressive and selective degeneration of neurons in distinct regions of the central nervous system (CNS).^1^, ^2^ A prominent characteristic of these disorders is the formation of protein aggregates.^3^ Characteristic aggregated proteins in NDDs encompass a spectrum of proteins, including amyloid precursor protein (APP, encoded by the APP gene), tau protein (encoded by the MAPT gene),^4^, ^5^ α‐synuclein (α‐syn, encoded by the SNCA gene),^5^, ^6^ TAR DNA binding protein 43 (TDP‐43, encoded by the TARDBP gene),^7^ dipeptide repeat proteins (DRPs, encoded by the C9orf72 gene),^8^ FUS RNA‐binding proteins (RBPs) (FUS, encoded by the FUS gene),^9^ Poly‐Q (encoded by the HTT gene),^10^ and cellular prion proteins (PrPC, encoded by the PRNP gene).^11^ The aggregation of these hallmark proteins is intricately linked to the pathogenesis of distinct neurodegenerative conditions. Consequently, this aggregation serves as a critical pathology foundation for both diagnosis of disease and therapeutic approaches. Furthermore, it has become a standard for diagnostic and classification purposes, applicable to a variety of neurodegenerative disorders, among them are Alzheimer's disease (AD),^12^ Parkinson's disease (PD),^13^ primary tauopathies (encompassing progressive supranuclear palsy [PSP],^14^ cortical basal ganglia degeneration [CBD],^15^ and tau‐linked frontotemporal dementia [FTD‐tau]^16^), FTD,^17^ amyotrophic lateral sclerosis (ALS), synucleinopathies (encompassing Lewy body dementia [LBD]^18^ and multiple system atrophy [MSA]^19^, ^20^), and Huntington's disease (HD).^21^

In the context of the 3D architecture and stability of proteins, those with inherent instability, particularly those abundant in beta‐folding,^22^, ^23^, ^24^ exhibit an increased propensity for aggregation.^25^ The mutation of proteins is a critical factor in aggregation, highlighting the significant role of protein overexpression in this process. Different mutations (eg, Swedish, Arctic, Dutch mutations) affect APP processing by increasing amyloid beta (Aβ) production or altering its properties.^23^, ^24^, ^25^ Additionally, the impairment of protein degradation is another factor contributing to protein aggregation, which is also a reason for changes in protein expression levels. Post‐translational modifications are crucial as well. Furthermore, gene mutations that alter protein–protein interactions can impact protein conformation changes and influence protein phase transitions, including liquid–liquid phase separation (LLPS). Proteins also exert their effects through intrinsically disordered regions (IDRs). Factors such as mutations,^26^ post‐translational modifications,^26^ and environmental conditions (eg, vibration,^27^ mechanical stress, temperature fluctuations, alterations in ionic strength, and pH of the surrounding milieu) can induce alterations in protein conformation. These changes may facilitate the transition of proteins from a soluble to an aggregated state, enhance the strength of inter‐protein interactions, and modulate interactions between molecular chaperones and proteins,^28^ which manifest as a reduction in molecular chaperone content or an elevation of protein concentration beyond the solubility threshold, thereby fostering aggregation into clusters.

**FIGURE 1:**
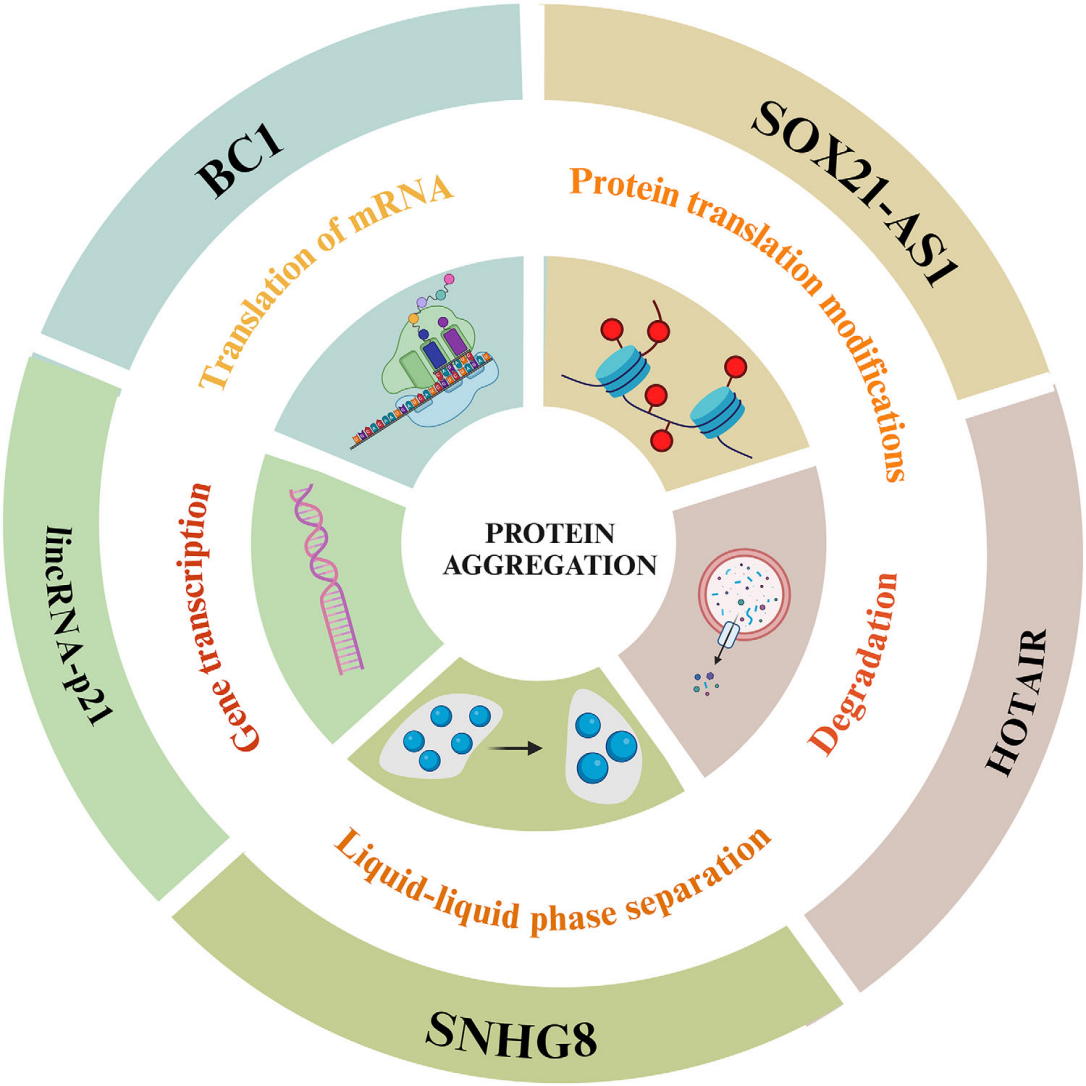
Schematic representation of neurodegeneration‐associated lncRNAs and their mechanisms of action. Examples of neurodegeneration‐related lncRNAs (outer ring). Classical pathways of neurodegenerative disorder, through which the lncRNAs act (middle ring). Neurodegeneration‐related lncRNAs can act in specific protein aggregation phase (center). Created with BioRender.com. lncRNA, long non‐coding RNA.

In general, four primary categories of key mechanisms impact protein aggregation. These encompass the promotion of protein overexpression by regulating gene transcription and translation,^26^, ^29^ the hindrance of protein degradation via lysosomal and autophagic pathways,^30^, ^31^, ^32^ abnormal modifications,^27^, ^33^ and phase transitions in proteins^28^, ^34^ to modulate the formation of protein aggregates^35^ (Figure 1).

## 2 STRUCTURE AND FUNCTION OF LNCRNAS

Long non‐coding RNAs (lncRNAs), once relegated to the category of “junk RNA” within the broader landscape of RNA molecules,^36^, ^37^ typically exceed a length of 200 base pairs (bp) and lack open reading frames (ORFs),^38^ thus precluding their involvement in protein synthesis.

In terms of structural and functional attributes, linear lncRNAs exhibit parallels with protein‐coding messenger RNAs (mRNAs), including similarities in sequence length, transcriptional dynamics, and post‐transcriptional regulation.^39^ Nevertheless, lncRNAs generally display shorter lengths, lower expression levels, and diminished sequence conservation compared to mRNAs. Emerging evidence underscores the involvement of lncRNAs in various disease processes, particularly in NDDS, where they are abundantly expressed and highly conserved in brain tissues.^40^

LncRNAs are key regulators of gene expression across pre‐transcriptional, transcriptional, and post‐transcriptional stages.^41^, ^42^, ^43^ They modulate gene activity through four distinct mechanisms: acting as signaling molecules, functioning as decoys, guiding transcriptional machinery, and providing scaffolding for molecular complexes.^39^ LncRNAs are specifically expressed in response to signals such as certain hormones, nutrients, or pathogens, thereby regulating downstream gene transcription and related gene expression.^44^, ^45^, ^46^, ^47^, ^48^, ^49^, ^50^ By employing these signals to control RNA for regulation, organisms achieve a more efficient response rate. This efficiency is due to the avoidance of protein translation, enabling a more rapid reaction to acute physiological changes.

LncRNAs can inhibit the interaction of proteins, miRNAs, or other RNA molecules with their target molecules through competitive binding. For instance, certain lncRNAs can sequester transcription factors, transcriptional co‐activators, or repressors, thereby preventing their binding to DNA and subsequent regulation of gene expression. Similar to Xist, TUG1 facilitates H3K27me3 modification by guiding the PRC2 chromatin remodeling complex to specific regions and competitively inhibiting EZH2 binding to the promoter regions of other genes, this process leads to specific chromosome inactivation and a reduction in trophoblast invasiveness.^51^, ^52^, ^53^ LncRNA PANDA has been shown to protect cells from apoptosis by interacting with the transcription factor NF‐YA, thereby inhibiting its binding to the promoters of apoptosis‐related genes.^54^, ^55^ Similarly, lncRNAs can function as “sponges” for miRNAs by binding to them, occupying the interaction sites between miRNAs and mRNAs, thereby blocking the inhibitory effects of miRNAs on their downstream target mRNAs. This indirect regulation of gene expression is known as the competing endogenous RNA (ceRNA) mechanism.^56^, ^57^

LncRNAs can also regulate gene expression by directing protein complexes, such as chromatin remodeling complexes and histone‐modifying enzymes, to specific DNA sequences.^58^, ^59^, ^60^, ^61^ This regulatory mechanism is mediated through interactions between lncRNAs and DNA, as well as potential interactions with DNA‐binding proteins.

LncRNAs also function as molecular scaffolds, facilitating the simultaneous binding of multiple protein or RNA molecules to form functional complexes. These lncRNAs possess multiple structural domains that enable interactions with various protein or RNA molecules concurrently, resulting in the formation of stable multiprotein complexes. For example, glycoLINC acts as a backbone for metabolon formation between all four glycolytic payoff phase enzymes along with lactate dehydrogenase A.^62^

Many lncRNAs multitask and can act simultaneously using all four of the previously mentioned methods. For example, HOTAIRM1 acts in different ways in the nucleus and cytoplasm.^63^

## 3 LNCRNAS PLAY A ROLE IN PROTEIN AGGREGATION

The involvement of lncRNAs is evident across various protein aggregation mechanisms and pathways discussed above. LncRNAs can directly participate in gene transcription regulation, leading to an upregulation of protein transcription associated with pathological processes. Additionally, proteins undergoing LLPS, such as FUS,^64^ TDP‐43,^65^ α‐syn,^66^ and tau,^67^ possess IDRs.^68^ IDRs closely link to prion‐like and low‐complexity structural domains (LCDs).^28^ Interactions between lncRNAs and these IDRs play a pivotal role in influencing protein aggregation, which gives rise to the phenomenon of LLPS,^69^, ^70^ facilitating the reversible partitioning of proteins and nucleic acids into micrometer‐scale liquid polymers with specific biologically relevant functions.^71^ LncRNAs promote phase separation through two distinct mechanisms. First, lncRNAs can interact with multiple proteins via their various structural domains, the secondary and tertiary structures of lncRNAs. For instance, the role of lncRNA NEAT1 in nucleolus formation is contingent on its interactions with multiple proteins.^72^ This domain‐specific binding allows for the precise spatial and temporal regulation of phase separation, exemplified by the role of Xist in X chromosome inactivation.^73^ Second, the negatively charged backbone of lncRNAs can be attracted to positively charged proteins, such as the interaction of lncRNA MALAT1 with nuclear proteins, which leads to the stabilization of phases within the nucleolus. Additionally, lncRNAs can interact with positively charged proteins in nuclear speckles to form stable phases.^74^, ^75^

Throughout the process of LLPS and protein overaccumulation, cells employ two principal mechanisms for the degradation of misfolded proteins. The first mechanism involves the ubiquitin‐proteasome system (UPS), primarily tasked with eliminating misfolded proteins in the form of monomers or smaller aggregates.^2^ The second pathway encompasses macroautophagy/autophagy,^76^ wherein protein aggregates or inclusion bodies are recruited into autophagosomes and subsequently translocated to vesicles/lysosomes for degradation.^77^, ^78^ LncRNAs possess the capability to impede or decelerate this degradation process.^79^, ^80^ Furthermore, lncRNAs play crucial roles in post‐translational modifications, exerting influence on epigenetic processes and regulating protein structure and function.^81^, ^82^, ^83^ It is noteworthy that certain modifications are associated with increased protein aggregation; however, not every modification at every site contributes to the phenomenon of protein aggregation.^84^, ^85^, ^86^

### 3.1 The role of lncRNAs in Aβ aggregation in AD

One of the pathological features of AD is the extracellular aggregation of Aβ.^87^ The diagnostic criteria for the early stage of AD via positron emission tomography‐computed tomography (PET‐CT) imaging encompass the visualization and quantification of Aβ deposits.^88^ The progression from preclinical to symptomatic AD appears to be correlated with a confluence of factors, including heightened Aβ concentrations,^89^ the maturation of aggregate formations,^90^ and the diffusion of deposits across various cerebral regions.^91^ Concurrently, the widely accepted amyloid cascade hypothesis posits Aβ as a principal instigator in the pathogenesis of AD, initiating modifications in tau proteins that subsequently set off a cascade of continuous responses throughout the progression of the disease.^92^, ^93^

**FIGURE 2:**
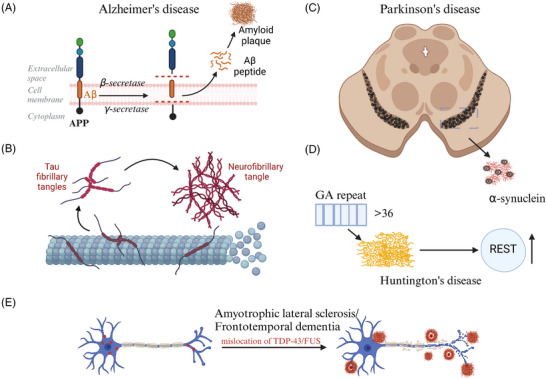
Diverse protein aggregates in neurodegenerative disorders. (A) in AD: In the amyloidogenic pathway of AD, APP is sequentially cleaved by BACE1 and gamma‐secretase complexes. This cleavage produces Aβ peptides of varying lengths. These Aβ monomers can spontaneously aggregate into amorphous oligomers, which further evolve into fibrillar aggregates characterized by an ordered structure, eventually forming amyloid plaques. (B) Tau protein aggregation in AD: In patients with AD, amorphous tau monomers, when hyperphosphorylated or interacting with other proteins, exhibit diminished affinity for microtubules. This reduction in binding capability facilitates the progressive formation of oligomers and ultimately culminates in the development of neurofibrillary tangles. (C) α‐syn in aggregation in PD: In PD, α‐syn within the nigral striatum forms oligomers as a result of external factors such as post‐translational modifications and proteolytic cleavage. These oligomers subsequently assemble into fibrillar structures enriched in β‐sheet formations. (D) Protein aggregation in HD: Overcopying of CAG repeat sequences, a manifestation of dipeptide repeat proteins (DRP) typically exceeding 36 repeats, within the HTT gene leads to an abnormal expansion of Poly‐Q sequences in the protein. This expansion fosters the formation of large inclusion bodies, contributing significantly to the pathology of HD. (E) Cytoplasmic mislocalization and aggregation of RBP in ALS and FTD: In ALS and FTD, the RBP FUS and TDP‐43 are mislocated to the cytoplasm due to mutations affecting their nuclear localization signals. The presence of LCDs in these proteins facilitates LLPS, leading to the formation of more stable, pathological aggregates. Created with BioRender.com. Aβ, amyloid beta AD, Alzheimer's disease; ALS, amyotrophic lateral sclerosis; BACE1, beta‐secretase; DRP, dipeptide repeat proteins; FTD, frontotemporal dementia; HD, Huntington's disease; LCDs, low‐complexity structural domains; LLPS, liquid–liquid phase separation; PD, Parkinson's disease; TDP‐43, TAR DNA binding protein 43.

The Aβ protein constitutes a soluble and amorphous peptide, originating from the APP, which undergoes sequential cleavage by β‐secretase enzyme (BACE1) and γ‐secretase enzyme, yielding various lengths of soluble Aβ fragments.^94^, ^95^, ^96^ Under the amyloidotic cleavage pathway, BACE1 initiates the cleavage of APP, leading to the generation of a 99‐amino‐acid C‐terminal fragment (C99) alongside a soluble fragment termed sAPP‐β.^97^ Subsequently, C99 undergoes further processing by the γ‐secretase complex, resulting in the production of Aβ peptides ranging from 37 to 43 amino acids in length,^96^, ^98^ with those peptides exceeding 40 amino acids displaying increased hydrophobicity and propensity for aggregation.^99^ The predominant forms of Aβ include Aβ40 and Aβ42, with monomeric Aβ displaying a proclivity toward oligomerization. These oligomers subsequently aggregate to form Aβ fibrils, commonly referred to as amyloid plaques (Figure 2B).

Senile plaques (SPs) represent extracellular accumulations of Aβ peptides. Prior investigations substantiated the existence of RNA within SPs,^100^ and an increased expression of non‐coding RNA has been identified in the cerebral tissue of individuals afflicted with AD,^101^, ^102^ which suggests that lncRNAs may be involved in the extracellular accumulation of Aβ. These lncRNAs act on Aβ aggregation in different ways (Table 1). LncRNAs possess the capacity to modulate the stability, localization, and translation of APP mRNA. Such as the primate‐specific BC200 RNA, along with its rodent counterpart BC1,^103^ represent evolutionarily conserved lncRNAs transcribed by RNA polymerase III.^104^ BC1 functions by facilitating the translation of APP mRNA via its interaction with fragile X syndrome protein (FMRP).^105^ This interaction underscores the pivotal involvement of BC1 in the regulatory processes governing Aβ production.

**TABLE 1:** Long non‐coding RNAs and related processes in Aβ.

LncRNA	Up‐Downregulation	Mechanism	Course	Reference
BC200	Up	Fragile X syndrome protein (FMRP)+BACE1	Influence on transcription and translation of APP	^105^, ^106^
BC1	Up	FMRP	‐	^105^
BACE1‐AS	Up	miRNA‐485‐5p/BACE1	‐	^107^
XIST	Up	miR‐124/BACE1	‐	^135^
51A	Up	SORL1	‐	^112^
H19	Up	miR‐15b/BACE1		^113^, ^114^
BDNF‐AS	Up	miR‐9‐5p/BACE1	‐	^110^
XIST	Up	EZH2/NEP	Degradation	^134^
LRP1‐AS	Up	High‐mobility group box 2 (Hmgb2)/LRP1	‐	^115^
NR2F1‐AS	‐	LRP1	‐	^116^
MIAT	‐	‐	‐	
KCNQ1OT1	‐	‐	‐	^117^
LUCAT1	‐	‐	‐	
FOXD2‐AS1	‐	miR‐143/LRP1	‐	^119^
NEATI	Up	PINK1+CAV2/TGFβ2/TGrDNI	‐	^121^, ^122^, ^126^
51A	Up	SORL1		^111^, ^112^
Sirt1‐as	‐	LXRα/ABCA1	‐	^4^, ^136^, ^137^
BACE1‐AS	Up	miR‐214‐3p/ (ATG5+LC3 I+LC3II+p62+Beclin‐1)	‐	^108^, ^138^

LncRNAs play a pivotal role in the generation of APP, while lncRNAs are instrumental in modulating the activity and quantity of the BACE1. For instance, a notable reduction in BC200 expression leads to a significant downregulation of BACE1 expression.^106^ The BACE1‐AS has been demonstrated to exert a positive regulatory influence on BACE1 expression via feedforward mechanisms.^107^ These mechanisms entail heightened BACE1‐AS expression subsequent to exposure to diverse cellular stressors, notably Aβ 1‐42. Additionally, XIST and BACE1‐AS are also involved in enhancing the stability of BACE1 mRNA through feedforward mechanisms.^107^, ^108^, ^109^ Moreover, exemplars such as 51A, H19, and BDNF‐AS have exhibited interactions with BACE1, further substantiating their involvement in these regulatory processes.^110^, ^111^, ^112^, ^113^, ^114^

The removal of soluble protofibrils and fiber forms of Aβ by glial cells occurs via two distinct pathways: inhibition of endocytosis and the inhibition of autophagy, respectively.

The antisense RNA linked with low‐density lipoprotein receptor‐related protein 1 (LRP1), denoted by LRP1‐AS, exhibits a direct binding affinity toward high‐mobility group box 2 (Hmgb2).^115^ Also, LRP1 plays a critical biological role in interactions with a variety of lncRNAs. These interactions impede Lrebp1a‐dependent transcription of LRP1, consequently interrupting LRP1‐mediated clearance of Aβ.^115^ Conversely, attenuation of NR2F1‐AS1 results in a direct inhibitory impact on the expression levels of LRP1 mRNA.^116^ Similarly, diminished levels of lncRNAs MIAT, KCNQ1OT1, LUCAT1, and FOXD2‐AS1 are associated with a reduction in LRP1 abundance.^117^, ^118^, ^119^

LncRNA NEAT1 engages in an interaction with NEDD4L, facilitating the PTEN‐induced ubiquitination and subsequent degradation of putative kinase 1 (PINK1).^109^ This process consequently disrupts the PINK1‐dependent mitochondrial autophagy/lysosomal pathway,^120^ culminating in the accumulation of Aβ.^121^ Additionally, NEAT1 modulates the transcriptional activity of CAV‐2,^122^ TGFβ2,^123^, ^124^ and TGFBR1.^122^ These three endocytosis‐related genes are epigenetically regulated by histone modifications, thereby suppressing the uptake and degradation of Aβ during endocytosis. Previous studies indicated a significant co‐expression of CAV‐1 and CAV‐2, with reduced CAV‐1 levels correlating with diminished α‐secretase activity and subsequent Aβ accumulation.^125^ However, recent findings suggest that alterations in CAV‐1 and CAV‐2 expression might not exert a notable influence on Aβ accumulation in AD.^126^ Moreover, TGFB2 has been implicated in auditory function,^127^, ^128^ and the downregulation of the lncRNA NEAT1 may lead to varied responses to acoustic stimuli, hinting at a potential association between hearing loss and AD risk mediated by lncRNAs.

Furthermore, the expression of 51A, situated within the intron 1 region of the SORL1 gene,^111^ orchestrates a splicing alteration in the SORL1 gene, leading to a transition from the synthesis of the conventional long protein SORL1 variant A.^129^ This transitional process consequently culminates in diminished synthesis of SORL1 variant A. SORL1 exerts its inhibitory effect on Aβ secretion through various mechanisms,^129^, ^130^ such as by binding to the substrate APP and impeding amyloid production within the trans‐Golgi network (TGN) vesicles.^111^, ^131^ Additionally, SORL1 demonstrates the capability to sequester newly synthesized Aβ in neurons and facilitate its degradation in lysosomes.^132^ Its interaction with apolipoprotein E (apoE) enables SORL1 to intervene in preventing pathological processes associated with phosphorylated tau (p‐tau) mislocation,^133^ thus implying that 51A may also mitigate p‐tau‐related pathological events.

The XIST exerts a depressant effect on the functioning of the Aβ‐degrading enzyme endoproteinase (NEP).^134^, ^135^ Sirt1‐as significantly increased the reverse cholesterol transport‐associated protein liver X receptor α (LXRα)^136^ and the ATP‐binding cassette transporter protein A1(ABCA1),^4^ which would leave Aβ reduced.^137^ Moreover, BACE1‐AS targets miR‐214‐3p, thereby upregulating ATG5 as a ceRNA, which is also implicated in the degradation of Aβ.^108^, ^138^ The Beclin 1/Vps34 complex is an Aβ crucial core component in the formation of autophagosomes and is known to be compromised in AD.^139^ Various lncRNAs have been demonstrated to interact with Beclin 1 in conditions such as pancreatitis,^140^ hepatocellular carcinoma,^141^ and myocardial dysfunction.^142^ These lncRNAs may also contribute to the clearance of Aβ and tau proteins.

In conclusion, the intricate regulatory roles of various lncRNAs highlight their significant involvement in modulating key pathways implicated in the clearance and accumulation of Aβ in AD. These lncRNAs influence processes ranging from Aβ degradation enzyme activity, endocytosis, autophagy, and mitochondrial function to alternative splicing events, underscoring their potential as therapeutic targets for combating AD pathology.

Peptide modifications such as glycosylation and phosphorylation are implicated in influencing both the production and deposition of Aβ. Similarly, these protein translational modifications (PTMs) exert a notable impact on the APP, affecting its cleavage in the course of pathological processes. Furthermore, the glycosylation,^143^ phosphorylation,^144^ and acetylation^145^ of BACE1 are pivotal in governing the transport and maturation of this enzyme. Progerin (PS), a significant constituent of the γ‐secretase complex, undergoes phosphorylation,^146^ thereby playing a regulatory role in the degradation of Aβ orchestrated by microglia. In addition, aberrant glycosylation of BACE1 hinders its targeting to the lysosome.^143^ Consequently, certain lncRNAs that are involved in the epigenetic regulation of BACE1 and PS may also influence the cleavage process of APP. Beyond these indispensable molecular players in Aβ production, it is noteworthy that glycosylation amplifies Aβ aggregation and subsequent toxicity.

The misfolding of extracellular Aβ peptides initiates the aggregation process, resulting in Aβ protofibrillarization. Consequently, toxic Aβ oligomers (AβOs) accumulate within the cerebral milieu of AD patients, serving as precursors for amyloid plaque deposition.^147^ AβO plays a pivotal role in orchestrating the assembly of amyloid protofibrils through LLPS, ultimately leading to the formation of replacement protofibrils. Furthermore, AβO functions as a receptor for the PrPC, inducing hydrogel formation upon binding. PrPC unfolds alpha helix Thr residues for liquid phase separation.^11^ Surprisingly, the involvement of lncRNAs in this LLPS‐mediated phase separation process, a pathway not considered a primary pathogenic mechanism for Aβ, remains unexplored.

### 3.2 The role of lncRNAs in tau aggregation in AD

The aforementioned assertion posited Aβ as the instigating factor; however, an additional prominent pathological manifestation in AD is the accumulation of insoluble tau inclusion bodies within the brain, predominantly manifesting as neurofibrillary tangles^148^ (Figure 2A). Mutations in the MAPT gene can also precipitate FTD, a condition characterized by the absence of amyloid plaques, a distinct clinical syndrome characterized by focal involvement of the prefrontal and anterior temporal lobes, is further categorized under tau pathology.^149^, ^150^ Specifically, types 3 and 4 of FTD are characterized by the hallmark features of tau‐positive neurofibrillary tangles or Pick‐like vesicles,^150^ which underscores the capacity of tau pathology to contribute to neurodegeneration independently of Aβ in AD. Under normal physiological conditions, tau proteins play a crucial role in microtubule polymerization and stabilization, a function that extends to their presence in other neurodegenerative disorders such as PD and corticobasal degeneration.^151^, ^152^ However, the accumulation of pathological tau species like neurofibrillary tangles can exert deleterious effects on neuronal function and viability, contributing to the neurodegenerative process in AD even in the absence of Aβ.^29^

Tau specifically interacts with genes or intergenic DNA sequences, with a preference for regions outside of the transcription start site ± 5000 bp and AG‐rich DNA sequences, with 30% of the interacting region overlapping with DNA sequences encoding lncRNAs.^153^ Prior investigations have extensively substantiated the existence of RNAs within neurofibrillary tangles,^154^, ^155^ serving as the principal non‐protein constituents of tau pathology and instigating the aggregation of tau proteins, ultimately culminating in the formation of paired helical fibers. Moreover, employing RNA sequencing techniques on cytoplasmic and nuclear tau aggregates in P301L mice and HEK293 cells has revealed a heightened presence of diverse lncRNAs within the protein aggregates.^154^ It has been reported that ncRNAs can influence the disease process by affecting gene expression and tau aggregation (Table 2). Notable among these lncRNAs are RMRP, NEAT1, XIST, MALAT1, TERC, LINC00630, SNHG19, LINC00342, and TUG1.^156^ This observation implies a pivotal role for lncRNAs in the intricate process of tau protein aggregation. However, the precise mechanism of lncRNAs such as LINC00630, TERC, and LINC00342, which are abundantly expressed in tau, remains inadequately elucidated within tau accumulation.^107^

**TABLE 2:** Long non‐coding RNAs and related processes in tau accumulation.

LncRNA	Up‐Downregulation	Mechanism	Course	Reference
MAPT‐AS1	Down	IRES/ribosomal RNA	Influence on transcription and translation of MAPT	^29^, ^157^
UBE3A‐ATS	‐	RBFOX1		^158^, ^159^
E230001N04Rik	‐	Sepk1/ Fkbp5		^171^
BACE1‐AS	Up	miR‐214‐3p/ATG5	Degradation	^108^
MAPT‐AS1	Up	ESCRT‐III	‐	^161^, ^162^, ^184^
Dubr	‐	YTHDF3	‐	^163^
HOTAIR	Up	Dzip3/Ataxin‐1+Mex3b/Snurportin‐1	‐	^164^
E230001N04Rik	‐	Sepk1/ Fkbp5	‐	^172^
RP11‐59J16.2	‐	MCM2	PTM	^181^
GAS5	‐	GSK‐3β /miR‐23b‐3p	‐	^183^
MAPT‐AS	‐	‐	‐	‐
BACE1‐AS	Up	‐	‐	^29^
SOX21‐AS1	‐	miR‐107		^185^
MEG3	Up	‐	‐	^186^
MALAT1	‐	miR144/Mtor/p‐tau	‐	^195^
KCNQ1OT1	‐	miR‐760/ CK2	‐	^117^
NEAT1	‐	FZD3/GSK3β/p‐tau	‐	^191^
NONMMUT055714	‐	GSK3β	‐	^189^
STEAP3‐AS1	‐	‐	‐	^187^
Mhrt779	‐	‐	‐	^188^
NEAT1	Up	miR‐15/107/CDK5R1	‐	^190^
HOTAIR	Up	‐	‐	‐
MALAT1	‐	miR144/p‐tau	‐	^195^

Two lncRNAs that have been more extensively characterized are MAPT‐AS1 and UBE3A‐ATS, which appear to regulate tau expression and aggregation through distinct pathways. The gene MAPT‐AS1 harbors an inherent mammalian extensive interpenetrating repeat (MIR), which functions to antagonize the internal ribosomal entry site (IRES) present within the MAPT mRNA, consequently impeding the translation process of tau protein through disruption of ribosomal RNA alignment.^157^ Furthermore, the lncRNA UBE3A‐ATS interacts with RBFOX1, thereby facilitating the association and stabilization of neuronal tau mRNA.^158^, ^159^ TDP‐43‐related lncRNAs act by regulating the function of TDP‐43, which in turn inhibits tau protein expression by increasing MAPT mRNA instability.^160^

The lncRNAs BACE1‐AS and MAPT‐AS1 exhibit distinct roles in cellular processes related to AD. BACE1‐AS modulates the autophagy pathway by binding to miR‐214‐3p, thereby regulating ATG5, a critical player in autophagosome formation.^108^ On the other hand, MAPT‐AS1 interferes with ribosomal RNA pairing, inhibiting tau translation and leading to increased MAPT mRNA levels.^157^ This elevation preserves the interaction between CHMP2 and CHMP4, preventing ESCRT‐III complex assembly.^161^, ^162^ Consequently, fusion between autophagosomes and lysosomes is impaired, compromising autophagic activity and promoting tau protein aggregation. The m6A‐modified long intergenic noncoding RNA (lincRNA) Dubr may have mitigated the reduction of tau by regulating the degradation of the RNA‐binding protein YTHDF3.^163^

In addition to the lncRNA‐mediated effects on autophagy and tau homeostasis, another lncRNA, known as HOTAIR, has also been implicated in the pathogenesis of AD. HOTAIR functions as both a scaffold and a promoter of protein ubiquitination.^164^ While its precise role in the degradation of tau remains unclear, the observation that HOTAIR levels are reduced in AD patients suggests that HOTAIR may contribute to the progression of AD by promoting protein ubiquitination.^165^

In AD, the levels and activity of Cdc25b are significantly elevated.^166^ This increase activates Cdk1/cyclin B, leading to the phosphorylation of tau and the FK506‐binding protein 51 kDa (FKBP51, encoded by FKBP5).^167^, ^168^, ^169^ FKBP51 forms a mature chaperone complex with Hsp90, which prevents tau degradation.^170^ The lncRNA E230001N04Rik promotes the expression of Cdc25B and SEPK1.^171^, ^172^ Other lncRNAs have been found to act on FKBP5 in breast cancer^173^ and nasopharyngeal carcinoma,^174^ suggesting that these lncRNAs may also play a role in the clearance of Aβ and tau.

PTMs of tau‐associated amino acid residues,^175^ encompassing isoasparagine formation, glycosylation, carbamoylation, N‐SUMOylation, proteolytic cleavage, C‐terminal truncation, partial pseudo‐phosphorylation, phosphorylation, and acetylation, collectively exert aggregation‐promoting effects.^176^, ^177^ Among these modifications, heightened phosphorylation of tau emerges as a pivotal and central factor, instigating conformational alterations that transmute the intrinsically unfolded tau into paired helical tau inclusion bodies and neuroprogenitor fiber tangles.

Emerging evidence suggests that lncRNAs also play a significant role in modulating tau pathology in AD.^178^, ^179^, ^180^ Several lncRNAs have been shown to influence the levels of p‐tau, a critical factor in the pathogenesis of AD. For instance, lncRNAs such as RP11‐59J16.2,^181^ GAS5,^182^, ^183^ MAPT‐AS,^161^, ^162^, ^184^ BACE1‐AS,^29^ SOX21‐AS1,^185^ and MEG3^186^ appear to elevate p‐tau abundance through diverse mechanisms. Conversely, lncRNAs including STEAP3‐AS1^187^ and Mhrt779^188^ have been observed to attenuate p‐tau quantities by modulating the activity of glycogen synthase kinase‐3β (GSK3β), a key regulator of tau phosphorylation. Intriguingly, the non‐coding RNA NONMMUT055714 exhibits a similar effect, decreasing p‐tau levels.^189^

The lncRNAs NEAT1 and HOTAIR have been shown to negatively regulate cyclin‐dependent kinase 5 regulatory subunit 1 (CDK5R1), a critical activator of CDK5, while MALAT1 exerts a positive regulatory effect on CDK5R1. Alterations in CDK5R1 levels directly influence CDK5 activity.^190^, ^191^ Overactivation of CDK5 leads to the pathological hyperphosphorylation of amyloid precursor proteins, tau proteins, and neurofilament proteins, thereby contributing to the progression of AD.^192^, ^193^ Furthermore, lncRNAs such as NEAT1^194^ and MALAT1^195^ promote tau protein phosphorylation by modulating microRNA expression, which further aggravates AD pathology.^196^, ^197^, ^198^ During AD pathogenesis, TDP‐43 and p‐tau exhibit a pathological synergy that exacerbates disease progression.^199^ LncRNAs, particularly NEAT1 and MALAT1, are implicated in this interaction,^200^, ^201^, ^202^ potentially modulating the crosstalk between TDP‐43 and p‐tau and thereby influencing the disease's molecular trajectory.

While phosphorylation of tau has long been recognized as a critical factor in the initiation of LLPs, emerging evidence suggests that other post‐translational modifications, such as acetylation, may also play a significant role in this process.

Acetylation of tau inhibits its degradation and contributes to tauopathy.^203^ While SIRT1‐mediated deacetylation and p300‐facilitated acetylation of tau have been well documented in the context of AD,^203^ the regulatory mechanisms governing these post‐translational modifications remain incompletely understood. Emerging evidence suggests that lncRNAs may play a crucial role in modulating the expression and activity of SIRT1, thereby indirectly influencing the balance of tau acetylation and deacetylation. While extant literature predominantly elucidates SIRT1's involvement in the progression of AD pathology through microRNAs, a comprehensive examination of lncRNAs influencing SIRT1 or p300 and subsequently modulating tau acetylation or deacetylation has not yet been conducted. Several lncRNAs, including GAS5, LincRNA‐p21, MCM3AP‐AS1, TUG1, SNHG7, SNHG8, SNHG10, SNHG15, Oip5‐as1, ILF3‐AS1, ANRIL, UCA1, and KCNQ1OT1, contribute to the regulation of SIRT1 expression by acting as miRNA sponges.^82^, ^133^, ^204^ These lncRNAs exert their influence on apoptosis, autophagy, oxidative stress, and inflammation, with observed alterations in their expression levels in diseased tissues, such as AD. Sequencing analyses reveal both increases and decreases in these lncRNAs, aligning with the trends observed in lesions leading to tau pathology.

The initiation of LLPS may be instigated either through the phosphorylation of individual tau proteins or through the involvement of RNA. Notably, the primary catalyst appears to be the interplay between tau proteins and RNA, with the resulting liquid condensates demonstrating superior characteristics in both microviscosity and thermal stability compared to hyperphosphorylated tau proteins.

The acetylation of tau proteins has been implicated in the direct facilitation of p‐tau protein accumulation, further contributing to the pathogenic cascade. In the context of LLPS, the acetylation of tau proteins is implicated in the direct facilitation of p‐tau protein accumulation. These p‐tau proteins exhibit a positive correlation with stress granule (SG) markers. Additionally, lncRNA SNHG8 demonstrates an affinity for RBPs, such as TIA1, FUS, DDX3X, and TDP‐43. Notably, a diminution in SNHG8 fosters the genesis of SGs associated with TIA1.^205^ These SGs, in turn, promote the phase separation of tau.

Conversely, PTM, specifically acetylation, mitigates the lysine‐driven liquid‐phase segregation. This attenuation stems from the amelioration of the positive charge on lysine residues within tau proteins. Such modulation confers neuroprotection by impeding the co‐localization of tau proteins with SGs. Consequently, this decelerates the LLPS of tau proteins. In this context, the aforementioned lncRNAs operate through the lncRNA‐PTM‐LLPS pathway, offering a comprehensive understanding of the interplay between these molecular components in neurobiological processes.

### 3.3 The role of lncRNAs in α‐syn aggregation in PD

The typical pathological manifestation of PD is characterized by the depletion of dopamine within the nigrostriatum and the accumulation of eosinophilic Lewy vesicles in the cytoplasm of the remaining nigrostriatal dopaminergic (DA) neurons. These Lewy vesicles, which are pivotal to the pathogenesis of PD, prominently feature the protein α‐syn as a key constituent. The aggregation of α‐syn is a critical process that underlies the development of Lewy body pathology and the subsequent neurodegeneration observed in PD (Figure 2C).

The aggregation of α‐syn can be delineated in three distinct stages. The initial, rate‐limiting step involves the conversion of soluble, unstructured monomeric α‐syn into partially soluble oligomers.

While genetic mutations in the SNCA gene, which encodes the α‐syn protein, have been well documented in PD, emerging evidence suggests that alterations in lncRNA levels may also play a significant role in the pathogenesis of this neurodegenerative disorder. Specifically, changes in lncRNA expression within the brain or in the periphery have been observed to coincide with SNCA gene mutations, raising the question of whether these lncRNA alterations may influence the accumulation and aggregation of α‐syn.^206^

The interaction between various lncRNAs and α‐syn deposition is multifaceted. First, in partnership with lncRNA G069488, transcripts RP11‐142J21.2 and AC009365.4 actively promote α‐syn deposition.^207^ Furthermore, MALAT1 enhances α‐syn stability through direct interaction.^208^ Similarly, lincRNA‐p21 serves as a molecular sponge for miR‐1277‐5p,^209^ thereby directly targeting SCNA. Concurrently, SNHG1 binds to miR‐15‐5p, leading to miR‐15‐5p downregulation and subsequent SNCA upregulation.^210^ Additionally, SNHG1 exacerbates α‐synaptic nucleoprotein aggregation through the miR‐15b‐5p/SIAH1 axis, causing toxicity.^211^ The heightened expression of NEAT1 not only triggers synaptonemal mRNA transcription but also results in their overexpression.^212^ Consequently, surplus α‐syn aggregates are internalized and transformed into highly folded Aβ protofibrils.^213^

In PD, the lncRNA UCHL1‐AS1 has been shown to play a crucial role in the regulation of α‐syn homeostasis. UCHL1, the protein encoded by the gene targeted by UCHL1‐AS1, is a deubiquitinase (DUB) that is closely associated with the ubiquitin‐dependent protein degradation pathway.^214^ By directly promoting the expression of UCHL1, UCHL1‐AS1 exerts a dual function in modulating α‐syn dynamics. Specifically, it elongates the Lys polyubiquitin chain on α‐syn, which prevents the proteasomal degradation of α‐syn. This mono‐ubiquitination stabilizes the monomeric form of α‐syn, making it less susceptible to proteasomal degradation. Additionally, ubiquitination impedes the fibrillation of α‐syn, thereby preventing its degradation. HTRA1 degrades α‐syn amyloidogenic fibrils, converting them into non‐toxic, seed‐incompetent species.^215^

Meanwhile, both HOTAIR and BDNF‐AS promote autophagy and apoptosis by abrogating microRNAs in PD.^216^, ^217^, ^218^, ^219^ L1CAM, identified as a neuron‐derived exosome associated with the autophagy‐lysosome pathway, along with the lncRNA linc‐POU3F3, actively regulates α‐synaptic nuclear proteins within the framework of L1CAM.^220^

The aggregation of α‐syn at the presynaptic terminus is a hallmark of PD pathology. Notably, various PTMs of α‐syn, such as SUMOylation, phosphorylation, nitration, O‐GlcNAcylation, and partial acetylation, have been shown to exert a significant influence on the structural transformations and aggregation propensity of this synaptic protein. However, the relationship between these PTMs and the potential involvement of lncRNAs in modulating α‐syn aggregation remains an area that requires further elucidation.

α‐Syn is composed of two primary regions: the membrane‐bound region and the acidic region. The membrane‐bound region includes the positively charged N‐terminal and the hydrophobic non‐amyloid component (NAC) regions. It is believed that the acidic region at the C‐terminal end interacts with the N‐terminal region, thereby providing protection to the NAC region and inhibiting LLPS,^66^ with PTMs exerting a notable influence on structural transformations analogous to those observed in the aggregation of synaptic nuclear proteins.^27^ Notably, SUMOylation, phosphorylation, nitration, O‐GlcNAcylation, and partial acetylation collectively contribute to the aggregation and resultant toxicity of α‐synaptic nuclear proteins. The relation of lncRNAs and PTMs remains to be defined.

Conformational changes of the intrinsically disordered protein α‐syn during LLPS involve a transition from a hairpin structure within the droplet to a more elongated conformational state.^66^ Nevertheless, the truncated carboxy‐terminal linkage structural domain of α‐syn exhibits a direct binding affinity to the proline‐rich region P2 of the tau protein.^213^, ^221^ This interaction is intricately regulated by the phosphorylation state of the tau protein.^213^ Consequently, α‐syn becomes notably concentrated within the separation droplets formed during tau phase separation. The Aβ plaque initiates the formation of a dynamic reservoir of pre‐misfolded α‐syn within dystrophic neuronal synapses. Proposing a potential involvement of lncRNAs in the LLPS of α‐syn underscores the significance of further elucidation.

Overall, a growing body of research suggests that lncRNAs play an important role in PD for α‐syn protein aggregation. These lncRNAs affect neuronal cell function and survival by regulating the process of α‐syn expression and aggregation. Therefore, therapeutic strategies targeting lncRNAs may provide new ideas to improve the symptoms and course of PD. Known lncRNAs associated with α‐syn aggregation are summarized in Table 3. Although there is currently no effective treatment for PD, in‐depth studies of lncRNAs’ function are expected to provide new targets for future interventions.

**TABLE 3:** Long non‐coding RNAs and related processes in Parkinson's disease.

lncRNA	Up‐Downregulation	Mechanism	Course	Reference
MALAT1	**‐**	β‐asarone	Influence on transcription and translation of SNCA	^210^
NEAT1	‐	‐	‐	^224^
lincRNA‐p21	‐	miR‐1277‐5p	‐	^211^
SNHG1	Up	miR‐15b‐5p/SIAH1	‐	^213^
UCHL1‐AS1	‐	UCHL1	‐	^225^
linc‐POU3F3	Up	LICAM	‐	^222^
BDNF‐AS	Up	microRNA‐125b‐5p, LC3II/I + Beclin‐1, p62	Degradation	^221^
HOTAIR	Up	miR‐221‐3p/ (LC3‐II/‐I+LAMP1/2+P62 + NPTX2)	‐	^218^, ^219^, ^220^

### 3.4 The role of lncRNAs in mHTT aggregation in HD

HD is a devastating neurodegenerative disorder caused by the expansion of trinucleotide CAG repeat sequences within the HTT gene. This genetic abnormality leads to the accumulation of Poly‐Q repeat sequences in the mHTT protein, which is a primary driver of the disease pathogenesis. As the number of Poly‐Q repeats increases beyond the normal range, the rate of amyloidogenic fibril formation and the subsequent aggregation of mHTT protein also rise significantly, contributing to the formation of protein deposits observed in the brains of individuals with HD (Figure 2D).

In the late stages of HD, NEAT1 levels are significantly elevated and closely associated with mHTT expression.^222^, ^223^, ^224^ This increase correlates with the facilitation of mHTT aggregation and also contributes to the impaired clearance of misfolded proteins. Moreover, the co‐localization of phosphorylated tau and HTT suggests the potential involvement of tau in the aggregation process of HTT protein.^225^

In HD, the mHTT protein undergoes extensive Poly‐Q repeat expansion, which leads to a decreased affinity for the repressor 1 silencing transcription factor (REST). This impaired interaction between mHTT and REST results in the excessive accumulation of nuclear REST,^226^ a crucial regulator of numerous neural‐specific genes and lncRNAs, many of which are hyper‐repressed in HD. The absence of nuclear REST has been observed in AD, FTD, and LBD, with its presence noted within autophagosomes alongside misfolded proteins. Consequently, lncRNAs that target REST could potentially contribute to the pathogenic processes of various NDDs.^227^ The dysregulation of this REST‐mediated transcriptional control is a key mechanism underlying the pathogenesis of HD.

Among these, the HAR1 lncRNA locus, comprising lncRNA HAR1F and lncRNA HAR1R transcripts, emerges as a direct target of REST.^228^ Additionally, discernible REST‐binding sites lie within 10 kb of the transcriptional start sites (TSSs) of several other lncRNAs, namely, LINC0341, RPS20P22 (a pseudogene of ribosomal protein S20), LINC00342, NEAT1,^229^ and MEG3,^224^ underscoring the intricate regulatory network involved in HD pathogenesis.

In conclusion, a growing number of studies have shown that lncRNAs have an important effect on HTT aggregation in HD (Table 4). These lncRNAs may play a key role in inhibiting or promoting HTT aggregation by regulating protein folding and aggregation processes. An in‐depth study of the regulatory mechanisms of lncRNAs on HTT aggregation in HD will help to unravel the pathogenesis of the disease and provide potential targets for the development of new therapeutic strategies.

**TABLE 4:** Long non‐coding RNAs and related processes in Huntington's disease.

LncRNA	Up‐Downregulation	Mechanism	Course	Reference
NEAT1	Up	‐	‐	^224^, ^225^, ^226^
TUG1	Up	‐	‐	^226^
HAR1F/ HAR1R	Down	miR‐124‐3	‐	^230^
LINC0341	‐	‐	mHTT	‐
RPS20P22	‐	‐	‐	^231^
LINC00342	‐	∖	‐	‐
MEG3	Down	‐	‐	^226^

### 3.5 The role of lncRNAs in RBP mislocation and aggregation in ALS and FTD

ALS and FTD are two devastating NDDs that share a common pathophysiological link in a subset of cases. A major common genetic factor contributing to both ALS and FTD is the expansion of GGGGCC in the C9orf72 gene,^8^, ^230^ which is believed to disrupt the normal function of the C9orf72 protein and lead to the accumulation of toxic RNA and dipeptide repeat proteins (DRPs).^231^ Additionally, both ALS and FTD are characterized by the presence of abnormal protein aggregates, such as TDP‐43^232^, ^233^, ^234^, ^235^ and FUS,^236^, ^237^ in the brains of affected individuals, suggesting the involvement of converging molecular pathways in the two disorders (Figure 2E).

C9ORF72‐AS can form RNA foci in the brain regions of ALS patients, such as the frontal cortex and cerebellum, as well as in the spinal cord of patients with ALS and FTD,^238^ which may be involved in the pathology of both diseases.

In the brains of individuals with FTD, the RBP TDP‐43 and FUS have been observed to co‐localize with the lncRNAs NEAT1_2^200^, ^222^ and MALAT1,^202^, ^239^ even in relatively healthy patients.^201^ This co‐localization represents one mechanism by which TDP‐43 and FUS regulate the transcription or transcriptional stability of lncRNAs. Dysregulation of lncRNAs occurs when functional TDP‐43 or FUS are depleted or unavailable.^240^ Additionally, the binding of lncRNAs to TDP‐43 and the binding of lncRNAs to FUS is essential for the proper execution of their cellular functions.^241^

This association suggests a potential involvement of these lncRNAs in the regulation of TDP‐43 and FUS, two key players in the pathogenesis of FTD and ALS.^242^ Notably, TDP‐43 has been shown to exhibit a preference for binding to extended UG‐rich sequences found in the lengthy intronic and 3′ untranslated regions (UTRs) of mRNA, further highlighting the complex interplay between RBPs and their RNA targets in these neurodegenerative disorders.^243^, ^244^

TDP‐43 and FUS are primarily RBPs soluble within the nucleus, where increased RNA concentrations act as buffers, hindering the phase separation of RBPs.^242^ The misplacement of these proteins to the cytoplasm could result in an overabundance of phase separation, leading to the formation of cytotoxic solid‐like assemblies.^201^, ^242^ This mislocalization may occur due to decreased nuclear RNA levels or the loss of RNA‐bound gene function.^242^ Consequently, changes in the composition and characteristics of subcellularly localized RNAs can modulate phase dynamics, thus counteracting or aggravating the pathological aggregation of RBPs. Phase separation of RBP in the cytoplasm depletes RBP in the nucleus and induces cell death.^245^

The purified structural domains of the C‐terminal region of TDP‐43 were found to induce aggregation, whereas elevated levels of NEAT1 in ALS were observed to promote in vitro LLPS of TDP‐43.^246^, ^247^, ^248^ The NEAT1‐mediated orchestration of TDP‐43 LLPS and the formation of droplet‐like nucleosomes led to an excessive cytoplasmic relocation of TDP‐43,^249^ resulting in the formation of SGs, a subset of RNA‐containing particles implicated in aberrant liquid‐to‐solid transitions within recombination chambers in vitro. Furthermore, cytoplasmic lesions of TDP‐43, undergoing phosphorylation under prolonged stress and damage to SGs,^72^ were found to compromise their RNA‐binding capacity, consequently facilitating the LLPS phenomenon of TDP‐43. Variants of SOD1 associated with ALS, which are characterized by misfolding, specifically accumulate and aggregate within the SGs of human cells. This accumulation adversely affects the dynamics of SGs, which are membrane‐less organelles composed of RBPs and RNA. Furthermore, these alterations in SG composition initiate an abnormal liquid‐to‐solid phase transition within recombinant compartments in vitro.^28^, ^250^ Notably, purified FUS also exhibited a propensity for aggregation; however, unlike TDP‐43, this trait may remain unaffected by mutations associated with ALS.^251^ Simultaneously, poly‐proline arginine (poly‐PR), identified as the most cytotoxic DRP in vitro, interacts with NEAT1, which serves as a structural scaffold, thereby exacerbating protein aggregation dynamics.^252^

In addition, MALAT1 plays a role in the formation of nuclear speckles, which are sites responsible for storing and modifying splicing factors.^253^ Meanwhile, Meg3 functions to downregulate the expression of TDP‐43. In a Drosophila model, knocking down the lncRNA CR18854 rescues impairments in climbing ability induced by dysregulation of Cabeza/Drosophila FUS (dFUS),^254^, ^255^ a direct homolog of human FUS.^256^ This dysregulation results in the depletion of dFUS, causing its translocation to the cytoplasm and subsequent loss of its nuclear function.

ALS and FTD often share genetic, pathological, and clinical features, indicating a close relationship. Abnormal protein aggregates like TDP‐43 and FUS are implicated in both disorders, in which lncRNAs act through the two binding proteins mentioned above (Table 5). Changes in RNA dynamics, including misplacement and aggregation of RBPs, contribute to disease progression. In particular, the lncRNAs NEAT1 and MALAT1 influence the formation of protein aggregates and cellular dysfunction. In a Drosophila model, manipulating lncRNA levels can mitigate neurodegenerative symptoms induced by protein dysregulation, highlighting potential therapeutic avenues.

**TABLE 5:** Long non‐coding RNAs and related processes in ALS/FTD.

lncRNA	Up‐Downregulation	Mechanism	Course	Reference
C9ORF72‐AS	‐	‐	‐	^240^
MALAT1	Up	‐	‐	^201^, ^202^, ^255^
Meg3	Down	‐	LLPS	^226^
CR18854	‐	dFIG4	‐	^256^
NEAT1	Up	DRP/FUS/TDP‐43	‐	^72^, ^250^

Abbreviations: ALS, amyotrophic lateral sclerosis; DRP, dipeptide repeat protein; FTD, frontotemporal dementia; TDP‐43, TAR DNA binding protein 43.

## 4 CONCLUSION AND PROSPECTIVE

The prevalence of NDDs is on the rise, increasingly positioning them at the forefront of public health discourse. There is compelling evidence to suggest that lncRNAs, acting as epigenetic regulators, play a significant role in the protein aggregation processes characteristic of these diseases. Crucially, lncRNAs influence not only the nature and functionality of the proteins involved but also impact processes such as LLPS and autophagy. Notably, hyperphosphorylated tau protein is implicated as a central component in the pathophysiology of virtually all NDDs.

However, current research into lncRNAs remains superficial. The majority of studies have focused primarily on accessible samples such as blood and exosomes, with mechanistic investigations largely centered on AD and PD. This indicates a pressing need for more in‐depth exploration and innovative breakthroughs. Given the potential for non‐coding regulatory elements to initiate cascading pathogenic effects, further studies should also extend to human induced pluripotent stem cell‐derived neurons, organoids, and neuropathological specimens.

Targeting lncRNAs within protein aggregates presents a novel therapeutic avenue for these otherwise intractable conditions, owing to their involvement in co‐regulatory pathways across different NDDs. This approach underscores their viability not only as distinctive biomarkers but also as promising therapeutic targets.

## CONFLICT OF INTEREST STATEMENT

The authors declare that they have no known competing financial interests or personal relationships that could have appeared to influence the work reported in this paper. Author disclosures are available in the Supporting Information.

## Supporting information



Supporting Information
