# Supplementary material for: Long non‐coding RNAs as key regulators of neurodegenerative protein aggregation
Source: Alzheimers Dement. 2025 Feb 12;21(2):e14498. doi: 10.1002/alz.14498 (PMC11815248; doi:10.1002/alz.14498)
Supplement: Supplementary file 1 — Supporting Information [file ALZ-21-e14498-s001.pdf]

ICMJE DISCLOSURE FORM

Date: 9/16/2024

Your Name: Qi Xu

Manuscript Title: lncRNAs as Key Regulators of Neurodegenerative Protein Aggregation

Manuscript Number (if known): ADJ-D-24-01780R1

In the interest of transparency, we ask you to disclose all relationships/activities/interests listed below that are related to the content of your manuscript. "Related" means any relation with for-profit or not-for-profit third parties whose interests may be affected by the content of the manuscript. Disclosure represents a commitment to transparency and does not necessarily indicate a bias. If you are in doubt about whether to list a relationship/activity/interest, it is preferable that you do so.

The author's relationships/activities/interests should be defined broadly. For example, if your manuscript pertains to the epidemiology of hypertension, you should declare all relationships with manufacturers of antihypertensive medication, even if that medication is not mentioned in the manuscript.

In item #1 below, report all support for the work reported in this manuscript without time limit. For all other items, the time frame for disclosure is the past 36 months.

		**Name all entities with whom you have this relationship or indicate none (add rows as needed)**	**Specifications/Comments (e.g., if payments were made to you or to your institution)**
**Time frame: Since the initial planning of the work**			
1	All support for the present manuscript (e.g., funding, provision of study materials, medical writing, article processing charges, etc.) **No time limit for this item.**	☒ None	
**Time frame: past 36 months**			
**2**	Grants or contracts from any entity (if not indicated in item #1 above).	☒ **None**	
**3**	Royalties or licenses	☐ **None**	
		Biorender	US27IJQSTW/AJ27IK4DZA

		Name all entities with whom you have this relationship or indicate none (add rows as needed)	Specifications/Comments (e.g., if payments were made to you or to your institution)
**4**	Consulting fees	☒ **None**	
**5**	Payment or honoraria for lectures, presentations, speakers bureaus, manuscript writing or educational events	☒ **None**	
**6**	Payment for expert testimony	☒ **None**	
**7**	Support for attending meetings and/or travel	☒ **None**	
**8**	Patents planned, issued or pending	☒ **None**	
**9**	Participation on a Data Safety Monitoring Board or Advisory Board	☒ **None**	
**10**	Leadership or fiduciary role in other board, society, committee or advocacy group, paid or unpaid	☒ **None**	

		Name all entities with whom you have this relationship or indicate none (add rows as needed)	Specifications/Comments (e.g., if payments were made to you or to your institution)
**11**	Stock or stock options	☒ **None**	
**12**	Receipt of equipment, materials, drugs, medical writing, gifts or other services	☒ **None**	
**13**	Other financial or non-financial interests	☒ **None**	

Please place an "X" next to the following statement to indicate your agreement:

☒ I certify that I have answered every question and have not altered the wording of any of the questions on this form.

ICMJE DISCLOSURE FORM

Date: 9/1/2024

Your Name: Ling-Qiang Zhu

Manuscript Title: lncRNAs as Key Regulators of Neurodegenerative Protein Aggregation

Manuscript Number (if known): ADJ-D-24-01780R1

In the interest of transparency, we ask you to disclose all relationships/activities/interests listed below that are related to the content of your manuscript. "Related" means any relation with for-profit or not-for-profit third parties whose interests may be affected by the content of the manuscript. Disclosure represents a commitment to transparency and does not necessarily indicate a bias. If you are in doubt about whether to list a relationship/activity/interest, it is preferable that you do so.

The author's relationships/activities/interests should be defined broadly. For example, if your manuscript pertains to the epidemiology of hypertension, you should declare all relationships with manufacturers of antihypertensive medication, even if that medication is not mentioned in the manuscript.

In item #1 below, report all support for the work reported in this manuscript without time limit. For all other items, the time frame for disclosure is the past 36 months.

	Name all entities with whom you have this relationship or indicate none (add rows as needed)	Specifications/Comments (e.g., if payments were made to you or to your institution)
Time frame: Since the initial planning of the work		

1	All support for the present manuscript (e.g., funding, provision of study materials, medical writing, article processing charges, etc.)<br>No time limit for this item.	☐ None<br><br>Hubei Provincial Natural Science Foundation (2022CFA004) — Ling-Qiang Zhu<br>Hubei Provincial Natural Science Foundation (2022RC4044, 2024JJ7442) — Ling-Qiang Zhu<br>Hubei Provincial Natural Science Foundation (23-44-00015). — Ling-Qiang Zhu<br>National Key R&D Program of China (2023YFE0117600) — Ling-Qiang Zhu<br>N.A.B. acknowledges financial support from RSF (23-44-00015) — Ling-Qiang Zhu<br>National Natural Science Foundation of China (82325017/ 82030032) — Ling-Qiang Zhu
		Time frame: past 36 months
2	Grants or contracts from any entity (if not indicated in item #1 above).	☒ **None**
3	Royalties or licenses	☒ **None**
4	Consulting fees	☒ **None**
5	Payment or honoraria for lectures, presentations, speakers bureaus, manuscript writing or educational events	☒ **None**
6	Payment for expert testimony	☒ **None**

**7**	Support for attending meetings and/or travel	☒ **None**
**8**	Patents planned, issued or pending	☒ **None**
**9**	Participation on a Data Safety Monitoring Board or Advisory Board	☒ **None**
**10**	Leadership or fiduciary role in other board, society, committee or advocacy group, paid or unpaid	☒ **None**
**11**	Stock or stock options	☒ **None**
**12**	Receipt of equipment, materials, drugs, medical writing, gifts or other services	☒ **None**
**13**	Other financial or non-financial interests	☒ **None**

Please place an "X" next to the following statement to indicate your agreement:

☒ I certify that I have answered every question and have not altered the wording of any of the questions on this form.

ICMJE DISCLOSURE FORM

**Date:**	9/16/2024
**Your Name:**	Dan Liu
**Manuscript Title:**	lncRNAs as Key Regulators of Neurodegenerative Protein Aggregation
**Manuscript Number (if known):**	ADJ-D-24-01780R1

In the interest of transparency, we ask you to disclose all relationships/activities/interests listed below that are related to the content of your manuscript. "Related" means any relation with for-profit or not-for-profit third parties whose interests may be affected by the content of the manuscript. Disclosure represents a commitment to transparency and does not necessarily indicate a bias. If you are in doubt about whether to list a relationship/activity/interest, it is preferable that you do so.

The author's relationships/activities/interests should be defined broadly. For example, if your manuscript pertains to the epidemiology of hypertension, you should declare all relationships with manufacturers of antihypertensive medication, even if that medication is not mentioned in the manuscript.

In item #1 below, report all support for the work reported in this manuscript without time limit. For all other items, the time frame for disclosure is the past 36 months.

		**Name all entities with whom you have this relationship or indicate none (add rows as needed)**	**Specifications/Comments (e.g., if payments were made to you or to your institution)**
		Time frame: Since the initial planning of the work	
1	All support for the present manuscript (e.g., funding, provision of study materials, medical writing, article processing charges, etc.) **No time limit for this item.**	☐ None	
		Hubei Provincial Natural Science Foundation (2023AFA068)	Dan Liu
		National Natural Science Foundation of China (82371403/ 82261138555/ 32070960)	Dan Liu
		Time frame: past 36 months	
**2**	Grants or contracts from any entity (if not indicated in item #1 above).	☒ **None**	
**3**	Consulting fees	☒ **None**	

		Name all entities with whom you have this relationship or indicate none (add rows as needed)	Specifications/Comments (e.g., if payments were made to you or to your institution)
**4**	Payment or honoraria for lectures, presentations, speakers bureaus, manuscript writing or educational events	☒ **None**	
**5**	Payment for expert testimony	☒ **None**	
**6**	Support for attending meetings and/or travel	☒ **None**	
**7**	Patents planned, issued or pending	☒ **None**	
**8**	Participation on a Data Safety Monitoring Board or Advisory Board	☒ **None**	
**9**	Leadership or fiduciary role in other board, society, committee or advocacy group, paid or unpaid	☒ **None**	
**10**	Stock or stock options	☒ **None**	

		Name all entities with whom you have this relationship or indicate none (add rows as needed)	Specifications/Comments (e.g., if payments were made to you or to your institution)
**11**	Receipt of equipment, materials, drugs, medical writing, gifts or other services	☒ **None**	
**12**	Other financial or non-financial interests	☒ **None**	
**13**	Other financial or non-financial interests	☒ **None**	

Please place an "X" next to the following statement to indicate your agreement: I certify that I have answered every question and have not altered the wording of any of the questions on this form.

☒

ICMJE DISCLOSURE FORM

Date: 10/28/2024

Your Name: Ying Su

Manuscript Title: lncRNAs as Key Regulators of Neurodegenerative Protein Aggregation

Manuscript Number (if known): ADJ-D-24-01780

In the interest of transparency, we ask you to disclose all relationships/activities/interests listed below that are related to the content of your manuscript. "Related" means any relation with for-profit or not-for-profit third parties whose interests may be affected by the content of the manuscript. Disclosure represents a commitment to transparency and does not necessarily indicate a bias. If you are in doubt about whether to list a relationship/activity/interest, it is preferable that you do so.

The author's relationships/activities/interests should be defined broadly. For example, if your manuscript pertains to the epidemiology of hypertension, you should declare all relationships with manufacturers of antihypertensive medication, even if that medication is not mentioned in the manuscript.

In item #1 below, report all support for the work reported in this manuscript without time limit. For all other items, the time frame for disclosure is the past 36 months.

	Name all entities with whom you have this relationship or indicate none (add rows as needed)	Specifications/Comments (e.g., if payments were made to you or to your institution)

Time frame: Since the initial planning of the work		
1	All support for the present manuscript (e.g., funding, provision of study materials, medical writing, article processing charges, etc.)<br>**No time limit for this item.**	☐ None<br><table><tr><td>Ministry of Science and Technology of the People's Republic of China (STI2030-Major Projects 2021ZD0201900)</td><td>Ying-Su</td></tr><tr><td></td><td></td></tr><tr><td></td><td>Click the tab key to add additional rows.</td></tr></table>
**Time frame: past 36 months**		
**2**	Grants or contracts from any entity (if not indicated in item #1 above).	☐ **None**
**3**	Royalties or licenses	☐ **None**
**4**	Consulting fees	☐ **None**
**5**	Payment or honoraria for lectures, presentations, speakers bureaus, manuscript writing or educational events	☐ **None**
**6**	Payment for expert testimony	☐ **None**

**7**	Support for attending meetings and/or travel	☐ **None**
**8**	Patents planned, issued or pending	☐ **None**
**9**	Participation on a Data Safety Monitoring Board or Advisory Board	☐ **None**
**10**	Leadership or fiduciary role in other board, society, committee or advocacy group, paid or unpaid	☐ **None**
**11**	Stock or stock options	☐ **None**
**12**	Receipt of equipment, materials, drugs, medical writing, gifts or other services	☐ **None**
**13**	Other financial or non-financial interests	☐ **None**

Please place an "X" next to the following statement to indicate your agreement:

☒ I certify that I have answered every question and have not altered the wording of any of the questions on this form.

ICMJE DISCLOSURE FORM

Date: 9/16/2024

Your Name: He-Zhou Huang

Manuscript Title: lncRNAs as Key Regulators of Neurodegenerative Protein Aggregation

Manuscript Number (if known): ADJ-D-24-01780R1

In the interest of transparency, we ask you to disclose all relationships/activities/interests listed below that are related to the content of your manuscript. "Related" means any relation with for-profit or not-for-profit third parties whose interests may be affected by the content of the manuscript. Disclosure represents a commitment to transparency and does not necessarily indicate a bias. If you are in doubt about whether to list a relationship/activity/interest, it is preferable that you do so.

The author's relationships/activities/interests should be defined broadly. For example, if your manuscript pertains to the epidemiology of hypertension, you should declare all relationships with manufacturers of antihypertensive medication, even if that medication is not mentioned in the manuscript.

In item #1 below, report all support for the work reported in this manuscript without time limit. For all other items, the time frame for disclosure is the past 36 months.

		Name all entities with whom you have this relationship or indicate none (add rows as needed)	Specifications/Comments (e.g., if payments were made to you or to your institution)
**Time frame: Since the initial planning of the work**			
1	All support for the present manuscript (e.g., funding, provision of study materials, medical writing, article processing charges, etc.) **No time limit for this item.**	☐ None	
		the National Natural Science Foundation of China (82471457,82001164)	He-Zhou Huang
**Time frame: past 36 months**			
**2**	Grants or contracts from any entity (if not indicated in item #1 above).	☒ **None**	
**3**	Consulting fees	☒ **None**	
**4**	Payment or honoraria for lectures, presentations, speakers bureaus, manuscript writing or	☒ **None**	

	educational events	
**5**	Payment for expert testimony	☒ **None**
**6**	Support for attending meetings and/or travel	☒ **None**
**7**	Patents planned, issued or pending	☒ **None**
**8**	Participation on a Data Safety Monitoring Board or Advisory Board	☒ **None**
**9**	Leadership or fiduciary role in other board, society, committee or advocacy group, paid or unpaid	☒ **None**
**10**	Stock or stock options	☒ **None**
**11**	Receipt of equipment, materials, drugs, medical writing, gifts or other services	☒ **None**
**12**	Other financial or non-financial interests	☒ **None**

**13**	Other financial or non-financial interests	☒ **None**

Please place an "X" next to the following statement to indicate your agreement: I certify that I have answered every question and have not altered the wording of any of the questions on this form.

☒
